# A Challenge in Perioperative Anesthetic Management: A Case Report of an Infant With Concurrent Ullrich Congenital Muscular Dystrophy and Pierre Robin Sequence

**DOI:** 10.7759/cureus.82170

**Published:** 2025-04-13

**Authors:** Aileen F Haque, Vidhi Patel, Victoria Bradford

**Affiliations:** 1 Anesthesiology, Wayne State University School of Medicine, Detroit, USA; 2 Pediatric Anesthesiology, Children's Hospital of Michigan, Detroit, USA

**Keywords:** anesthetic management, anticipated difficult airway, congenital airway malformation, pediatric-anesthesia, pediatric difficult airway, pediatrics, pierre robin sequence, ullrich congenital muscular dystrophy

## Abstract

Ullrich congenital muscular dystrophy (UCMD) is a severe form of progressive congenital muscular dystrophy, and it presents with severe hypotonia, recurrent respiratory infections, respiratory failure, and problematic airway management secondary to congenital anatomical abnormalities and high aspiration risk. Pierre Robin sequence (PRS) is a rare congenital syndrome presenting at birth with a triad of micrognathia, glossoptosis, and chronic upper airway obstruction leading to known difficult airway management and opioid sensitivity. Perioperative management of both UCMD and PRS requires careful consideration of airway management, choice of anesthetic agents, and potential involvement of respiratory complications; each condition presents its own unique challenges. This report discusses the successful, complete anesthetic management of a patient with collagen 12 (COL12A1)-variant UCMD and concurrent PRS.

## Introduction

Ullrich congenital muscular dystrophy (UCMD) is a severe form of congenital muscle dystrophy that presents with severe hypotonia in the neonatal period [1,2]. It is exceedingly rare, with only 50 registered molecularly-confirmed cases worldwide so far. A majority of the known cases of UCMD are due to mutations in the collagen 6 gene; up until 2017, there had been no documented cases of patients with the collagen 12 (COL12A1) gene mutation [3]. Perioperative management of UCMD patients requires careful consideration of airway management, choice of anesthetic agent, and potential involvement of respiratory complications, as many of them are prone to recurrent respiratory infections [1,2,4]. Studies have shown success with the use of propofol and opioids in combination as a total intravenous anesthetic in these patients, thereby avoiding the use of volatile agents. It is important to avoid volatile anesthetic agents in patients with muscular dystrophies, as these patients are predisposed to rhabdomyolysis and hyperkalemia, in addition to having an increased risk of developing an asystole heart rhythm [1,2]. Pierre Robin sequence (PRS) is a congenital birth defect mainly characterized by upper airway obstruction, cleft palate, glossoptosis, and micrognathia, with an incidence ranging from 1 in 5000 to 1 in 85000 [5,6]. Careful preoperative anesthetic planning regarding airway management with a multidisciplinary team is warranted in PRS patients due to the known significant airway deformities. We present a unique case of the successful anesthetic management of an infant with concurrent COL12A1-variant UCMD and PRS.

Informed consent was obtained from the patient's legal guardian before the preparation of this report. This article was previously presented as a poster at the 2024 Society of Pediatric Anesthesiologists Annual Meeting on April 13, 2024.

## Case presentation

**Figure 1:**
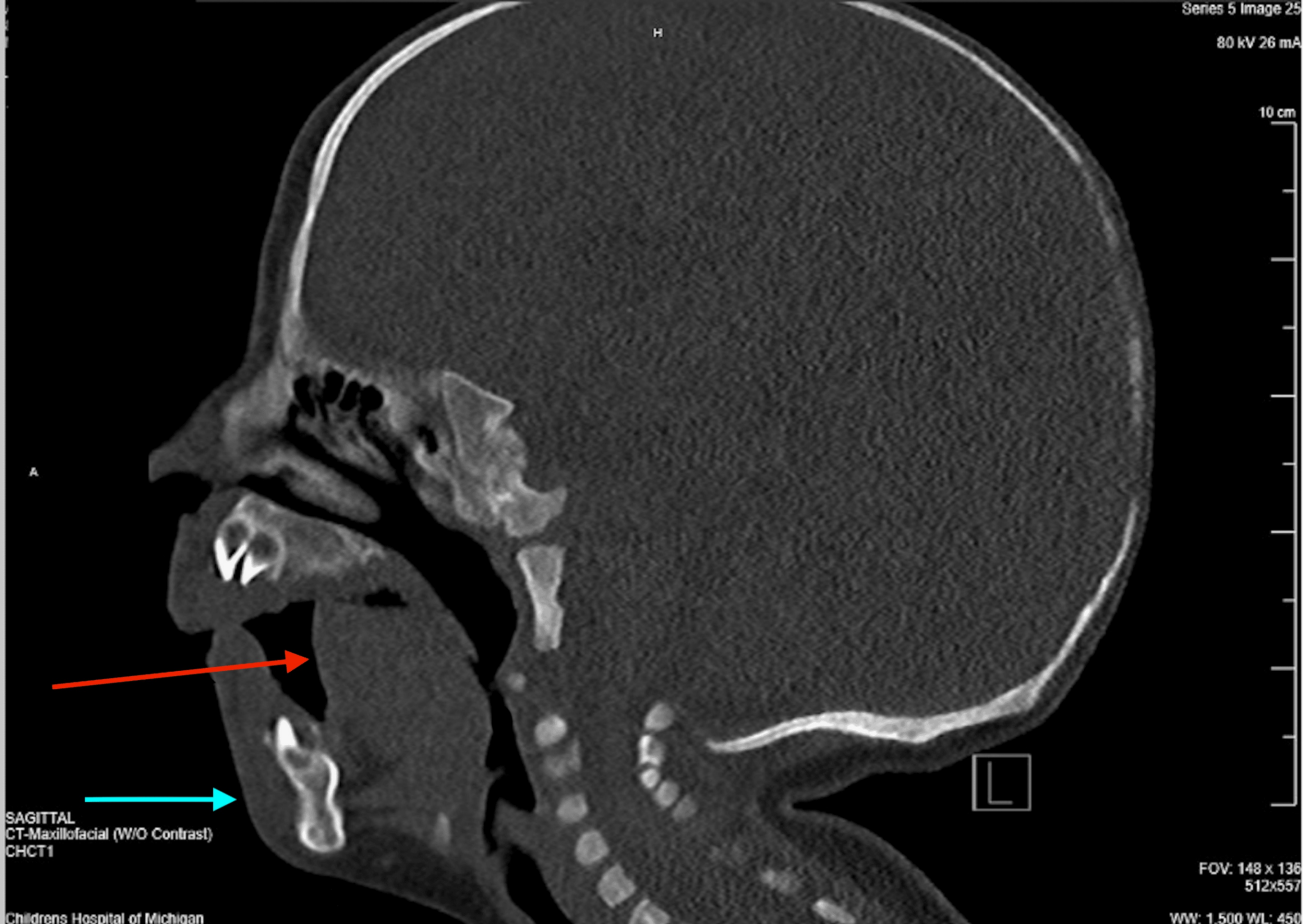
Sagittal view of the patient’s maxillofacial CT scan The image demonstrates the prominent micrognathia (blue arrow) and severe glossoptosis (red arrow) causing severe obstruction of the patient’s airway CT: computed tomography

A nine-month-old, 8.23-kg male presented for an elective laparoscopic first stage orchiopexy and circumcision due to bilateral non-palpable testes found incidentally on prior circumcision evaluation. The patient had a known history of hypotonia in the setting of UCMD with confirmed COL12A1 gene homozygous mutations. He also had PRS and laryngomalacia diagnosed on maxillofacial CT imaging (Figure 1), airway endoscopy, direct laryngoscopy, and bronchoscopy before this surgery. In addition to the above findings, the patient presented with the following issues: recurrent respiratory infections, severe obstructive sleep apnea with nocturnal 0.25 L oxygen requirements diagnosed by a sleep study, placement of a gastrostomy, glossoptosis, micrognathia, dolichocephaly, sialorrhea, and scaphocephaly.

Upon being brought to the OR, the anesthesia team was initially unsuccessful in obtaining intravenous (IV) access while the patient was awake due to a history of poor vasculature. After multiple attempts, 1.0 MAC of sevoflurane was administered for a short period to assist with obtaining IV access. Once IV access was obtained, the inhalational agent was turned off immediately, and the following medications were administered before endotracheal intubation: 30 mg of propofol, 15 mcg of fentanyl, and 5 mg of rocuronium.

As anticipated, given his history of UCMD and PRS, the patient had a difficult airway due to the presence of the following congenital abnormalities: limited mouth opening, micrognathia, glossoptosis, palate with a high V-shaped arch in the midline, and a short neck. Two-person mask ventilation was required with two skilled providers to achieve successful mask ventilation. Initially, direct laryngoscopy was attempted, and there was no obtainable laryngoscopic view. Subsequently, video laryngoscopy was utilized with a Glide 2.5 Lo Pro blade to obtain a grade III view and successful endotracheal tube placement. The patient was found to have an anterior larynx as well with the placement of the endotracheal tube.

Once the airway was secured, maintenance of anesthesia was achieved using total IV anesthesia (TIVA) with a propofol infusion running at 200 mcg/kg/min and carefully titrated 5 mcg fentanyl and 2 mcg dexmedetomidine boluses as needed for analgesia, limiting opioid use throughout the case. No additional neuromuscular blocking agent or inhalation agents were used during the case. The orchiopexy and circumcision procedures were completed without difficulty by the urology team. Throughout the procedure, the patient had hemodynamically stable vitals and no signs of ECG changes consistent with hyperkalemia. On emergence, the propofol infusion was turned off, and the rocuronium was reversed with 20 mg of sugammadex. The patient was extubated awake successfully to 3 liters of oxygen via nasal cannula in the operating room before being taken to the recovery area. 

Due to the patient's complex respiratory history, he was admitted overnight for observation. He was weaned down to nocturnal home oxygen requirements overnight, had appropriate pain control, and had no postoperative anesthetic complications before discharge from the hospital the next morning.

## Discussion

Effective preoperative planning by the perioperative team in both airway management and choice of anesthetic agents utilized during the procedure resulted in an optimal outcome in our patient. First, there were multiple anatomical considerations taken into account that led to this patient having an anticipated difficult airway. Due to the established UCMD, the patient presented with limited mouth opening, contractures and stiffness of the cervical spine, respiratory muscle insufficiency leading to increased risk of respiratory failure, recurrent respiratory infections, and reduced airway patency [1,2,3]. Secondary to PRS, the patient presented with micrognathia, glossoptosis, airway obstruction, baseline obstructive sleep apnea (OSA) with nocturnal home oxygen requirements, and laryngomalacia [5,6]. However, due to the patient’s age, he was unable to cooperate with the providers. Therefore, despite having an anticipated difficult airway, awake intubation techniques were not attempted due to the lack of cooperation, and an asleep airway management plan had to be executed by utilizing the known pediatric difficult airway algorithms, as it is a common problem in the difficult pediatric airway [6,7].

Secondly, studies have shown that the choice of primary anesthetic is crucial in patients with UCMD and PRS due to specific contraindications related to the conditions and for avoiding adverse side effects. Third, patients with UCMD are at a risk of high aspiration due to the baseline hypotonia leading to weakening of the muscles required for swallowing. Aspiration leads to an increased risk of postoperative complications ranging from hypoxia to acute respiratory distress syndrome (ARDS) to death in a patient already at a baseline higher risk for respiratory failure secondary to respiratory muscle insufficiency and chronic upper airway obstruction.

Multiple sources have shown that using TIVA with propofol and opioid analgesia is successful in UCMD patients, allowing avoidance of volatile anesthetics, which predispose some patients with muscular dystrophies to rhabdomyolysis, hyperkalemia, and asystole [1,2,3,8]. Some studies have shown that children with Ullrich’s myopathy, a milder form of the disease, do not develop rhabdomyolysis with volatile anesthetic usage, which is the rationale for inducing the patient with sevoflurane for a few minutes to obtain IV access after the multiple unsuccessful attempts leading to lack of infant cooperation; however, as a precaution, the TIVA was initiated as soon as IV access was obtained to prevent any of the risks described above that are associated with muscular dystrophies [4,9]. Rather than making an opioid infusion part of the TIVA, fentanyl boluses were given as needed with careful consideration of the patient’s history of severe OSA [5].

Although caudal blocks are known to decrease opioid requirements [1], this was deferred in our patient due to an active diaper rash and subsequent infection risk. Adding in a regional or neuraxial anesthetic component to the anesthetic plan would decrease overall general anesthetic requirements and provide improved analgesia while allowing for the reduced use or possibly total avoidance of opioid usage, a benefit for PRS patients with severe OSA.

Finally, while there is controversy regarding the use of neuromuscular blockade in patients with muscular dystrophies due to their baseline hypotonia and the concern for postoperative respiratory failure, full reversal with sugammadex leads to spontaneous ventilation and successful extubation without postoperative respiratory complications, as demonstrated in our patient [9]. A literature review revealed no documented reports of the complete anesthetic management of patients with COL12A1-variant UCMD and concurrent PRS.

## Conclusions

We reported a successful case of the complete anesthetic management of a patient with COL12A1-variant UCMD and concurrent PRS. Several studies have described the anesthetic management of PRS patients, and a few studies have discussed the anesthetic management of UCMD patients in the literature. However, there are many key differences in terms of implementing a successful anesthetic between UCMD and PRS patients based on congenital birth defects and resulting medical complications of each respective condition, which will continue to require further research. Therefore, as highlighted in this case, the development and execution of a safe and effective tailored anesthetic for pediatric patients with concurrent UCMD and PRS is much more complex and requires extensive preoperative planning for success.

## References

[1] Puangsuvan N, Mester RA, Ramachandran V, Tobias JD (2009). Perioperative care of a child with Ullrich congenital muscular dystrophy. Middle East J Anaesthesiol.

[2] Radeka JZ, Stojanovic MD, Vasilijic MN, Randjelovic MM, and Jankovic RJ (2023). Anesthesia and rare neuromuscular diseases. Front Anesthesiol.

[3] Punetha J, Kesari A, Hoffman EP (2017). Novel Col12A1 variant expands the clinical picture of congenital myopathies with extracellular matrix defects. Muscle Nerve.

[4] Grosu I, Truong D, Teodorescu S, Mousny M, Veyckemans F (2012). Anesthetic management of a child with Ullrich myopathy. J Anesth.

[5] Cladis F, Kumar A, Grunwaldt L, Otteson T, Ford M, Losee JE (2014). Pierre Robin sequence: a perioperative review. Anesth Analg.

[6] Sinkueakunkit A, Chowchuen B, Kantanabat C (2013). Outcome of anesthetic management for children with craniofacial deformities. Pediatr Int.

[7] Krishna SG, Bryant JF, Tobias JD (2018). Management of the difficult airway in the pediatric patient. J Pediatr Intensive Care.

[8] Vandenberghe W, Jacobs TF, Plasschaert FS, Willems J, Den Blauwen NM, Vereecke HE, Wouters P (2010). Anesthesia and perioperative management for a patient with Ullrich syndrome undergoing surgery for scoliosis. Acta Anaesthesiol Belg.

[9] Erbabacan E, Köksal GM, Şeker TB, Ekici B, Özcan R, Altindaş F (2015). Anaesthesia management and use of sugammadex in a patient with Ullrich's disease. Turk J Anaesthesiol Reanim.

